# Perceived Well-Being and Quality of Life in People with Typical and Atypical Development: The Role of Sports Practice

**DOI:** 10.3390/jfmk5010012

**Published:** 2020-02-13

**Authors:** Massimo Ingrassia, Francesco Mazza, Piera Totaro, Loredana Benedetto

**Affiliations:** Department of Clinical and Experimental Medicine, University of Messina, 98125 Messina, Italy

**Keywords:** sports practicing, perceived well-being, quality of life, general self-efficacy, satisfaction with life, disability

## Abstract

Sports and physical activity are recognized as a source of psycho-physical well-being. Therefore, practicing sports can improve the perception of the quality of life (QoL). The study investigated in adults with atypical development (AD) and typical development (TD) if the perceptions of well-being and QoL may depend on the frequency of practicing sports. Participants were 51 AD (age *M* = 32.98, *SD* = 7.81; 45.1% female) and 270 TD adults (age *M* = 26.50, *SD* = 6.52; 79.3% female) subsequently divided into three groups: 1) people not practicing sports, or 2) practicing it occasionally, or 3) practicing it regularly. We adopted general self-efficacy, life satisfaction, positive well-being, and psychological distress related to physical exercise as measures of well-being, WHOQOL-BREF as QoL, and I-TIPI-R as indicators of Big Five personality factors. Questionnaires were completed online. Practicing sports influenced the perception of well-being and QoL. For the AD group, sporting practice seems to have assumed an equalization effect, eliminating the statistical differences between well-being and QoL measures of AD and TD groups. Associations emerged between Big Five and well-being and QoL indices with some differences as a function of group characteristics. In summary, results suggest that practicing sports is strongly associated with improvements in perceptions of well-being and QoL, especially in disabled persons.

## 1. Introduction

Practicing sports is an expanding phenomenon integrated to contemporary culture, to which people associate fundamental values for individual development. Indeed, it seems practicing sports is an essential factor to develop personal resources and increase specific skills, such as self-consciousness, confidence in own abilities, autonomy, etc. [1,2]. A reason for this widespread practice of sports probably lies in people’s desire to improve their own *quality of life* (QoL), a purpose of primary interest to be pursued for the current society [3].

In the Nineties, the World Health Organization (WHO) defined QoL as ’a person’s perception of his/her position in life within the context of the culture and value systems in which he/she lives and in relation to his/her goals, expectations, standards, and concerns. It is a broad-ranging concept incorporating, in a complex way, the person’s physical health, psychological state, level of independence, social relationships, personal beliefs and their relationships to salient features of the environment’ [4]. The theoretical model derived from nearly 15 years of international research and experimentation outlines QoL as a multi-dimensional profile across 25 sub-domains (named *facets*) grouped in six *domains* (physical health, psychological, levels of independence, social relationships, environmental, and spirituality, plus overall QoL and general health perceptions). Facets describe behaviors (e.g., activities), states of being (e.g., fatigue), capacities (e.g., the ability to move around), or subjective perceptions of experiences (e.g., pain); domains describe core aspects of QoL cross-culturally defined [5]. Therefore, WHO’s QoL is a construct based on individuals’ concerns rather than on the conceptualizations of medical professionals: subjective perceptions and judgments are crucial. Everyone tends to develop a personal vision of what is a good or a bad QoL, taking into account his/her own health conditions, personality, and styles of interactions with the limits and the opportunities offered by the environment [6]. 

The perception of a better QoL is positively associated with the performance of physical activities and sports [7,8]. However, this is not the only positive association. Indeed, the practice of physical activities is related to the improvement of health-related physical fitness (including components associated with health promotion or disease prevention, such as cardiorespiratory fitness, strength and muscular resistance, body flexibility, etc.) and with academic performances [9,10]. Furthermore, sporting activity offers the individual the possibility of expressing physical and relational abilities and resources he/she may not know they have because they have never expressed them before. Therefore, sports would take on the role of facilitators of the recovery of unexpressed functions and personal autonomy [11].

Serious threats to QoL are limitations in activities and restrictions to participation, that is, the two conditions that together with impairments and in interaction with environmental and personal factors determine a disability [12]. Just in the field of disability studies, Schalock and colleagues [13,14] proposed an ecological model of QoL with eight core domains (i.e., emotional well-being, interpersonal relations, material well-being, personal development, physical well-being, self-determination, social inclusion, and rights) where autonomy and social relationships are crucial (for a review, see [15]). Some studies [9] have emphasized that people with disabilities who practice sports activities develop greater self-confidence and increase their self-esteem, as well as the ordinary performances of daily life activities are faced with greater well-being [16]. Other studies show that participation in sports and recreational activities is undoubtedly a benefit for people with disabilities [17,18]. Facing new challenges of sporting activity, the individual may enhance his/her skills for action and social participation, increasing the self-perception of positive control over external events. In this sense, sport would also become a crucial element for the individual’s motor, mental, relational, and emotional development [9].

Disability is the consequence of a plurality of factors which in a specific person mark a significant deviation from the expected functioning for that person at that age and in that context of life. Furthermore, disability can also derive from a disease condition without tissue or organ impairments. Therefore, since just as WHO points out [12, Annex 5] any term used to name the “person with disability” can also involuntarily become discriminatory, we prefer the terms “with atypical development” and “with typical development” to refer to people with and without disabilities, respectively.

Sporting activity has a significant impact also on person’s *life satisfaction* due to the feeling that the person has about his/her own physical form, considered synonymous with good health [11]. QoL and life satisfaction are two concepts strictly connected: both are subjective judgments about the quality of one’s life experiences and both require a multidimensional assessment (i.e., the estimation of physical and motor, psychological or mental, emotional, relational, and contextual indices). WHOQOL indices reflect the multidimensional complexity of construct, that cannot be averaged in a single measure as “life satisfaction” or others [19]. However, people can also produce overall judgments about their lives that, even if referring to different experiential facets and dimensions, are formulated not as an average or a sum of the single facets but as a global judgment that concerns the whole *life satisfaction*, like when they are asked if they are happy [20]. Sports practicing may influence QoL and life satisfaction [11]. Therefore, it can be affirmed that sporting activity affects the perceived degree of life satisfaction and, if it is regularly practiced, it is positively important for physical and mental health and QoL [16,17,18].

The main purpose of this study is to investigate whether the perceptions of well-being and QoL of people with atypical and typical development significantly differ as a function to their engagement in sports activities. The selected measures of well-being are indicators of life satisfaction, general self-efficacy, and positive well-being and psychological distress for physical exercise. QoL is measured in four domains (i.e., *physical health*, *psychological health*, *social relationships*, and *environment*) with the WHOQOL-BREF. The hypotheses to test are: a) people practicing sports refer more favorable perceptions of QoL and well-being than people not practicing sports; b) people with atypical development and practicing sports have a significant reduction of the gap in measure differences from people with typical development and practicing sports. 

A second objective has an exploratory nature and aims to estimate which personality characteristics (i.e., Big Five) are associated with practicing or not practicing sports.

## 2. Materials and Methods 

### 2.1. Participants

Participants were recruited through a non-probabilistic online sampling procedure: on thematic sites and blogs where we posted a selection notice about the general scope of the study and the ways to participate in it. We searched adults aged between 20 and 45 years with functional disabilities, i.e., with health conditions that could induce a limitation of an activity or a restriction on participation [12]. Exclusion criteria were age below/above 20–45 years and the need for assistance in completing the questionnaires for lack of understanding compilation instructions. Fifty-one people affirmatively responded and completed the questionnaires. They declared the following health conditions: 22 persons (43.1%) motor disability, 10 (19.6%) chronic illness, 9 (17.7%) psychological disorders, 8 (15.7%) deafness, and 2 (3.9%) intellectual disability. These participants were included in the “Atypical Development group” (AD; age *M*_ad_ = 32.98, *SD*_ad_ = 7.81; 45.1% female).

Later, with a snowball online procedure, we recruited people with no health problems and aged between 20 and 45 years. There were 271 who affirmatively responded and completed the questionnaires and we named them the “Typical Development group” (TD; age *M*_td_ = 26.50, *SD*_td_ = 6.52; 79.3% female). Final participants were 321. Table 1 reports participants’ general frequencies (gender, marital status, education, and annual income).

### 2.2. Measures and Procedure

Participants completed:

*(i)*  An ad hoc checklist to collect the main personal data and information about health conditions and sports activity practiced.
*(ii)*  *General Self-Efficacy Scale*—GSE [21]: The scale was created to assess a general sense of perceived self-efficacy with the aim to predict coping with daily hassles as well as adaptation after experiencing all kinds of stressful life events. It consists of 10 items evaluated on a 4-point Likert scale, from 1(*not at all true*) to 4 (*exactly true*), and it yields a total score between 10 and 40. There is no cut-off score to categorize persons. The Cronbach’s alphas obtained in various researches were greater than 0.85 [22,23]. In this study, alpha was 0.91.
*(iii)*  *Satisfaction with Life Scale*—SWLS [20]: A 5-item scale designed to measure global cognitive judgments of one’s life satisfaction. Participants indicate how much they agree or disagree with each of the 5 items using a 7-point scale that ranges from 7 (*strongly agree*) to 1 (*strongly disagree*). SWLS has shown reliability coefficients ranging between 0.79 and 0.89 [24]. In this study, alpha was 0.90.
*(iv)*  *The Subjective Exercise Experiences Scale*—SEES [25]: A measure of global psychological responses to the stimulus properties of exercise. It consists of 12 items evaluated on a 7-point Likert scale from 1 (*not at all*) to 7 (*very much*). It consists of 3 sub-scales, each of 4 items: *Positive well-being* (PWB), *Psychological distress* (PD), *Fatigue* (FAT). In this study, the alpha coefficients were good (0.78, 0.87, and 0.87, respectively), almost equal to ones of the original validation, i.e., 0.86 for PWB, 0.85 for PD, and 0.88 for the *Fatigue* sub-scale [25].
*(v)*  *World Health Organization Quality of Life*—WHOQOL-BREF [19]: It is the 26-item short form of the WHOQOL-100 and assesses 24 facets of QoL grouped in four domains that by structural equation modeling revealed the grouping solution to be more appropriate [26]. Domains are physical health (7 items), psychological health (6 items), social relationships (3 items), and environment (8 items), plus 2 items about overall QoL and general health. In this study, alpha coefficients were good enough (0.72, 0.61, 0.66, and 0.75, respectively), quite similar to ones (range 0.65 to 0.80) in the Italian validation study [27].
*(vi)*  *Ten-Item Personality Inventory* (I-TIPI–R) [28], the revised Italian version of the original scale by Gosling and coll. [29]: In ten items, that is, only 2 items for each of the five factors, it measures the classic Big Five personality dimensions. Each of the ten items is rated on a 7-point scale ranging from 1 (*strongly disagree*) to 7 (*strongly agree*). In this study, internal consistency indices of scales were lower than those in the original Italian validation [28] (Extraversion, α = 0.62; Agreeableness, α = 0.19; Conscientiousness, α = 0.43; Neuroticism, α = 0.54 [we used the reverse score as Emotional stability]; Openness, α = 0.29), but as Gosling and coll. [29] affirmed, “Cronbach’s alpha is a function of the mean inter-item correlation and the number of items comprising the scale. (…) with only two items per scale, the TIPI instead emphasized content validity consideration, resulting in lower inter-item correlation than is typical of a more homogenous scale.” (p. 516). The I-TIPI–R is useful for time-limited contexts or large survey questionnaires [28].

Respondents filled online the questionnaires after giving informed consent to the processing of personal data, according to the Italian legislation on the protection of personal data.

### 2.3. Data Analyses

Data were processed with IBM SPSS Statistics for Windows 22.0. Reliability (Cronbach’s alpha) and group differences (*F* test by ANOVA and MANOVA) were estimated for each scale. Subsequently, we evaluated measure associations by calculating Pearson’s *r* coefficients, differentiating the participants according to group conditions: i.e., AD and TD and if they do or do not practice sports.

## 3. Results

All measures were found to be reliable enough, with the Cronbach’s α coefficients included between 0.61 (psychological health of WHOQOL) and 0.91 (GSE). The five I-TIPI-R scales, whose coefficients α ranged from 0.19 (agreeableness) to 0.62 (extraversion), were a special case, as mentioned above.

A filter question (‘Do you practice any sporting activity?’) with three alternative answers allowed participants to be divided into three subgroups, i.e., those who play sports “never”, “occasionally”, and “regularly”: AD = 25.5%, 23.5%, and 51.0%, and TD = 21.1%, 37.0%, 41.9%, respectively. So, we tested group differences through a series of factorial designs 2 (AD vs. TD) × 3 (Sports practice: Never, Occasionally, and Regularly), with the scale scores as dependent variables.

### 3.1. Group Differences

*GSE Scale*: As we hypothesized, participants who practice sports on average had higher levels of GSE (*F*[2, 315] = 9.52, *p* < 0.001, η_p_² = 0.06). No differences emerged between the AD group vs. TD group (*F*[1, 315] = 2.81, *p* = 0.10, η_p_² = 0.01), but the interaction “Group × Sports practice” was significant (*F*[2, 315] = 4.74, *p* = 0.009, η_p_² = 0.03). Indeed, a more detailed examination of contrasts and pairwise comparisons revealed very interesting group differences. It should be noted the different trends of the two groups’ (AD vs. TD) mean scores: they are very different for those who do not practice sports in favor of the TDs (*t*[315] = −3.24, p = 0.001), but they are not statistically different for those who practice sports both occasionally and regularly (see Table 2 for descriptive statistics). Furthermore, contrast analyses showed that GSE index failed producing significant differences among TD participants as a function of sports practice (*F*[2, 315] = 1.81, *p* < 0.17, η_p_² = 0.01), but the differences are significant for the AD group (*F*[2, 315] = 8.19, *p* < 0.0001, η_p_² = 0.05).

*SWLS:* Participants practicing sports obtained higher average scores (*F*[2, 315] = 5.75, *p* = 0.004, η_p_² = 0.04). A Helmert contrast, with “never” condition as control, revealed a significant difference versus the other two conditions (*t*[315] = −3.13, *p* = 0.002), but not between “occasionally” and “regularly”. Neither differences emerged between the AD group vs. TD group (*F*[1, 315] = 0.14, *p* = 0.71, η_p_² = 0.0004), nor the interaction "Group × Sports practice" was significant (*F*[2, 315] = 0.77, *p* = 0.47, η_p_² = 0.005).

*SEES indices:* A 2 (Group) × 3 (Sports practice) MANOVA design, with PWB, PD, and FAT indices as dependent variables, was executed. Multivariate tests produced significant results for “Group” (λ = 0.96, *F*[3, 313] = 5.75, *p* = 0.002, η_p_² = 0.05) and “Sports practice” (λ = 0.94, *F*[6, 626] = 5.75, *p* = 0.005, η_p_² = 0.03), but not for the interaction “Group × Sports practice” (λ = 0.99, *F*[6, 626] = 0.79, *p* = 0.58, η_p_² = 0.008). The between-subjects tests revealed: (*i*) significant “Group” differences for PWB (*F*[1, 315] = 5.56, *p* = 0.019, η_p_² = 0.02) and FAT (*F*[1, 315] = 11.73, *p* = 0.001, η_p_² = 0.04), but not for PD (*F*[1, 315] = 3.53, *p* = 0.061, η_p_² = 0.01); (*ii*) significant “Sports practice” differences for PWB (*F*[2, 315] = 3.76, *p* = 0.024, η_p_² = 0.02), PD (*F*[2, 315] = 8.98, *p* < 0.0001, η_p_² = 0.05), and FAT (*F*[2, 315] = 4.32, *p* = 0.014, η_p_² = 0.03); and finally, (*iii*) no significant result in “Group × Sports practice” interactions. The Helmert contrasts applied to “Sports practice” with “never” as control vs. other two conditions highlighted *t*(315) = −2.54, *p* = 0.011; *t*(315) = 3.44, *p* = 0.001; and *t*(315) = 2.10, *p* = 0.036, for PWB, PD, and FAT, respectively; but no significant result between “occasionally” and “regularly” comparisons.

*WHOQOL physical health, psychological health, social relationships, and environment indices:* A 2 (Group) × 3 (Sports practice) MANOVA design, with WHOQOL indices as dependent variables, was executed (see Table 3 for descriptive statistics of WHOQOL indices). We observed significant results in multivariate tests for “Group” (λ = 0.77, *F*[4, 312] = 23.22, *p* < 0.0001, η_p_² = 0.23) and “Sports practice” (λ = 0.88, *F*[8, 624] = 5.06, *p* < 0.0001, η_p_² = 0.06), but not for the interaction “Group × Sports practice” (λ = 0.98, *F*[8, 624] = 0.75, *p* = 0.65, η_p_² = 0.009). The between-subjects tests revealed: (*i*) significant “Group” differences for *physical health* (*F*[1, 315] = 40.51, *p* < 0.0001, η_p_² = 0.11), but not for *psychological health* (*F*[1, 315] = 2.88, *p* = 0.091, η_p_² = 0.009), *social relationships* (*F*[1, 315] = 0.60, *p* = 0.806, η_p_² = 0.0002), and *environment* (*F*[1, 315] = 2.14, *p* = 0.144, η_p_² = 0.007); (*ii*) significant “Sports practice” differences for *physical health* (*F*[2, 315] = 17.38, *p* < 0.0001, η_p_² = 0.10), *psychological health* (*F*[2, 315] = 13.24, *p* < 0.0001, η_p_² = 0.08), *social relationships* (*F*[2, 315] = 4.65, *p* = 0.01, η_p_² = 0.03), and *environment* (*F*[2, 315] = 10.23, *p* < 0.0001, η_p_² = 0.06); and finally, (*iii*) no significant result in “Group × Sports practice” interactions.

The Helmert contrasts applied to “Sports practice” with “never” as control vs. the other two conditions highlighted *t*(315) = −5.37, *p* < 0.0001; *t*(315) = −4.60, *p* < 0.0001; *t*(315) = −2.77, *p* = 0.006; and *t*(315) = −3.54, *p* < 0.0001, for *physical health*, *psychological health*, *social relationships*, and *environment*, respectively; but no significant result between “occasionally” and “regularly” comparisons.

### 3.2. Correlational Analysis

In performing correlational analysis, we removed the *Fatigue* index of SEES, because its utility is probably linked to a rehabilitative use of the physical exercise [25], but not to strict evaluation of well-being connected to exercise, that is estimated by PWB and PD as its positive and negative poles, respectively. Furthermore, we grouped participants in four classes: with atypical development and practicing sports (AD-S), with typical development and practicing sports (TD-S), with atypical development not practicing sports (AD-NS), and with typical development not practicing sports (TD-NS). Participants practiced sports occasionally and regularly have been put together. Table 4 and Table 5 reports Pearson’s coefficients: S and NS, respectively; below the diagonals there are the AD groups, above the diagonals the TD groups.

As expected, GSE and SWLS measures significantly correlate positively with WHOQOL indices and PWB score and negatively with the PD score for groups of participants practicing sports. Some differences should be noted between the AD-S and AD-NS subgroups and between the AD and TD groups in the ways in which the well-being measures, WHOQOL indices, and I-TIPI-R scores correlate. An example is an association between SWLS and WHOQOL *social relationships*, that is, significantly positive for the AD-S group but slight and not significant for the AD-NS group. Another example is the WHOQOL *physical health* index significantly associated with I-TIPI-R scores, with the exception of the Openness factor, in the TD-S group but not in the AD-S group.

## 4. Discussion

The principal scope of this research was to prove people practicing sports, especially if atypically developed, have higher levels of well-being and QoL perceptions than people not practicing sports. This happened for all indices we had chosen for measuring well-being and QoL.

In these adult participants, the grade of GSE perception seems to benefit from sporting practice only for the AD group. Indeed, people with TD showed similar grades of GSE regardless of the condition of sports practice. However, if comparing ADs *vs.* TDs, only the “never” condition of sports practice reveals significant differences with a higher grade expressed by TDs. For the other two conditions, an equalization effect seems to happen: no difference emerged in comparisons of AD *vs.* TD groups. For the other indices of well-being (i.e., SWLS and SEES scales), we observed the same above equalization functions of practicing sports. In AD conditions, SWLS increases for those practicing sports up to exceed the corresponding average score of TD participants, and globally no difference emerges between AD and TD conditions. Furthermore, AD and TD together show a significant progression from “never” to “regularly” condition of practicing sports, therefore the sports practice seems useful for increasing the life satisfaction of people with both typical and atypical development. The SEES indices we considered (i.e., PWB and PD) showed a trend similar to SWLS in the conditions of sports practice, but in this case, the AD group *vs.* TD group comparison revealed significant differences with AD participants highlighting a better than average score in the PWB scale. Once again, we are witnessing an equalization phenomenon (according to the spirit of the UN’s *Standard Rules* [30]). 

The above results suggest that sporting practice can greatly influence people’s perceptions of their general self-efficacy, life satisfaction, positive well-being, and psychological distress, especially if they are atypically developed.

We observed a similar effect of equalization also in the QoL results. AD and TD groups showed significantly different average scores only for *physical health* scale, but no other AD *vs.* TD group differences emerged for *psychological health*, *social relationships*, and *environment* scales. Participants of both AD and TD groups who practiced sports “occasionally” or “regularly” always exhibited higher levels of perceived QoL than AD and TD groups not practicing sports. 

The *physical health* domain refers to evaluations about pain, medical treatments, energy for everyday life, mobility, sleeping quality, daily living abilities, and working capacities. Practicing sports does not seem to influence the AD *vs.* TD group evaluation of the *physical health* domain: the perception gap between the two groups remains the same regardless of the frequency of sports practice. However, if we consider sports activity regardless of developmental conditions (AD and TD), practicing sports favors an increase in perceptions of *physical health*.

Other domains refer to: estimation about enjoying life, living a meaningful life, concentration abilities, accepting bodily appearance, being satisfied with his/herself, and frequency of negative feelings (i.e., *psychological health*); estimation about satisfaction with personal relationships, sex life, and support by friends (i.e., *social relationships*); and estimation about feeling safe in daily life, healthiness of the physical environment, having enough money to meet needs, availability of daily information, having opportunity of leisure activities, satisfaction with the condition of living place, access to health service, and transport (i.e., *environment*). For these domains, we do not observe gaps in perceptions between AD *vs.* TD group comparisons (i.e., results are different if compared to that observed for *physical health*). However, if we consider sports activity regardless of developmental conditions (AD and TD), we observe again that practicing sports favors an increase in perceptions of these domains of QoL. Indeed, the frequency of practicing sports seems to be the only factor affecting QoL perception related to *psychological health*, *social relationships*, and the *environment*.

These results appear globally relevant and consistent with the data present in literature, according to which the practice of sporting activity reduces psychological distress to the advantage of better well-being and QoL, especially for disabled persons [31,32,33,34].

Finally, some considerations are to be made about the associations between personality characteristics and practicing/not practicing sports. First, Agreeableness and Openness reveal very negligible relations with all well-being and QoL indices, with the exception of Openness-GSE association for AD-not practicing sports group showing a high positive correlation.

Second, Extraversion displays low or negligible relations with well-being and QoL indices of both AD and TD groups practicing sports. For not-practicing-sports groups, Extraversion results *weakly* but significantly positively related to WHOQOL *psychological health*, GSE, and SEES-PWB, and negatively to SEES-PD in the TD group; in the AD group, Extraversion shows considerable correlations with WHOQOL *psychological health*, GSE, and SEES-PD, but they are not statistically significant perhaps because of the small number of group participants. Moreover, this group shows an anomalous high negative correlation value between Extraversion and WHOQOL *social relationships*, that no other group highlights.

Third, Conscientiousness displays associations similar to those already highlighted for Extraversion in practicing-sports groups, i.e., weakly or very negligible relations. For the Ad-not practicing-sports group, correlations are significantly high with WHOQOL *physical* and *psychological health*, and SWLS; it is very high with GSE. For the TD-not-practicing-sports group, Conscientiousness correlations are absolutely negligible.

Lastly, Emotional stability displays positive considerable relations with all well-being and QoL indices in the TD-practicing-sports group; positive considerable relation with GSE and positive but law coefficients with WHOQOL *physical* and *psychological health*, and SEES-PW in the AD-practicing sports group. The associations in not-practicing-sports groups are different: positive and low with WHOQOL *social relationships* and SWLS, high and positive with WHOQOL *psychological health*, but negative with SEES-PD in TD participants; correlations are high and positive with WHOQOL *physical health*, GSE, SWLS, and SEES-PWB, considerable and negative with SEES-PD, and finally weak and positive with WHOQOL *psychological health* in AD participants.

Overall, taken together, correlations do not exhibit a clear pattern of associations either in the four subgroups or in the two practicing/not practicing sports aggregations. Therefore, new empirical data become necessary.

## 5. Conclusions

The strength of sports is that they are activities facilitating the consolidation of self-efficacy and personal empowerment, characteristics that are not limited to the competitive contexts but usually are also generalized to other life contexts. By preparing his/herself for a constant both physical and mental commitment, each can improve Self-concept, because through sporting activity, the person can have the satisfaction of achieving set goals [35]. Although practicing sports is generally considered beneficial to health, many people find it is difficult to maintain a high level of physical activity, especially due to lack of motivation or knowledge [36]. Being able to choose between different sports should facilitate autonomy and the development of intrinsic motivation for engaging in a physically active lifestyle, through which they can experience psychosocial benefits [37]. Furthermore, one should not overlook the fact that intrinsically rewarding activities are more likely to be re-proposed [38]. Consequently, if a choice is offered that satisfies the needs of the person to a greater extent in terms of autonomy, competence and relationships, he/she will most likely grow in self-determination, learning, and well-being [35,39].

Despite its contribution to understanding the link between sports and quality of life, the study has some limitations. One of these concerns the characteristics of the groups: the participant numbers were not homogeneous between the AD and TD groups, nor for those practicing sports nor for those not practicing sports. Furthermore, the AD group was considered as a unit without taking into account the type of disability (as they do, for example, [40,41]). A second limitation concerns the type of sporting activity practiced: it was not taken into account whether it was individual or group, competitive or amateur. Future studies should take into account these additional sources of variability in assessing the perceptions of quality of life in relation to sports practice. Finally, a further limitation is to have chosen to recruit the participants and carry out the collection of answers via the web. This can prove to be complex for some potential participants, who find it difficult to interact with technological devices and are automatically excluded from the recruitment.

However, although these limitations exist, the study confirms that sport can be a fundamental experience in people’s lives. In fact, through practicing sports and physical activity, developing people have the opportunity to increase their self-esteem [42], social participation, and perceptions of well-being and quality of life. From an educational point of view, this evidence is important. As Murphy and coll. wrote [43] (p. 1057) ‘the participation of children with disabilities in sports and recreational activities promotes inclusion, minimizes deconditioning, optimizes physical functioning, and enhances overall well-being’. Therefore, these facts are meaningful stimuli to promote educational programs to enhance and empower typically-and-atypically-developed children’s participation in sports and physical activities. 

## Figures and Tables

**Table 1:** Participants’ characteristics.

Socio-Demographic Features	People with Atypical Development *n* = 51	People with Typical Development *n* = 270
**Gender**		
Male	28	56
Female	23	214
**Marital status**		
Unmarried	33	234
Married	14	32
Divorced	3	4
Widowed	1	0
**Education**		
Primary school	2	0
Secondary school	11	3
High school	23	102
Bachelor	6	111
University degree	9	54
**Annual income (euros)**		
≤5000	3	27
5001–15,000	12	68
15,001–30,000	19	57
30,001–45,000	5	26
45,001–60,000	2	13
>60,000	1	5
Rather not answer	4	28
I don’t know	5	46

**Table 2:** Descriptive statistics of *General Self Efficacy Scale* (GSE), *Satisfaction with Life Scale* (SWLS), and *The Subjective Exercise Experiences Scale* (SEES).

Scale	Sports Practice	Atypical Development *n* = 51			Typical Development *n* = 270		
		*M* (*SD*)	Skewness	Kurtosis	*M* (*SD*)	Skewness	Kurtosis
GSE – (10 items; α = 0.91)	Never	29.08 (7.38)	1.18	1.63	35.84 (6.02)	−0.27	−0.11
	Occasionally	39.08 (8.39)	−0.99	1.12	37.03 (6.25)	−0.33	0.37
	Regularly	37.12 (8.57)	−0.73	0.64	37.93 (6.96)	−0.28	−0.20
SWLS – (5 items; α = 0.90)	Never	17.85 (4.10)	1.13	2.17	19.42 (7.76)	−0.04	−1.10
	Occasionally	23.08 (6.86)	0.47	−0.86	21.02 (7.07)	−0.25	−0.47
	Regularly	23.39 (7.17)	−0.03	−0.84	22.64 (6.48)	−0.51	−0.30
SEES – PWB (4 items; α = 0.78)	Never	16.54 (4.61)	1.47	1.96	15.65 (4.11)	−0.02	−0.49
	Occasionally	19.42 (4.17)	−0.31	−1.03	16.77 (4.89)	0.20	−0.34
	Regularly	19.69 (5.45)	−0.11	−0.87	17.57 (5.37)	−0.20	−0.27
SEES – PD (4 items; α = 0.87)	Never	12.08 (4.66)	0.14	−1.17	13.49 (5.74)	0.42	0.32
	Occasionally	9.25 (5.28)	0.63	−1.06	11.24 (6.11)	0.86	0.12
	Regularly	7.39 (5.15)	2.19	4.97	9.16 (5.53)	1.48	1.88
SEES – FAT (4 items; α = 0.87)	Never	14.39 (4.94)	0.50	−0.48	19.58 (6.29)	−0.64	−0.46
	Occasionally	14.25 (5.68)	−0.80	0.15	17.08 (5.48)	−0.15	−0.19
	Regularly	12.89 (7.31)	0.60	−0.83	14.73 (5.94)	0.23	−0.64

Note. PWB = Positive Well-Being; PD = Psychological Distress; FAT = Fatigue.

**Table 3:** Descriptive statistics of the *World Health Organization Quality of Life—BREF* questionnaire.

Scale	Sports Practice	Atypical Development *n* = 51			Typical Development *n* = 270		
		*M* (*SD*)	Skewness	Kurtosis	*M* (*SD*)	Skewness	Kurtosis
Physical health (7 items; α = 0.72)	Never	18.15 (2.94)	−0.11	−1.49	23.54 (3.96)	−0.06	−0.51
	Occasionally	22.67 (2.84)	−0.25	1.02	25.42 (4.14)	−0.39	0.36
	Regularly	23.23 (5.40)	−0.22	−0.98	27.01 (3.29)	−0.18	−0.17
Psychological health (6 items; α = 0.61)	Never	17.23 (2.86)	0.18	−1.50	17.75 (4.18)	−0.51	0.36
	Occasionally	21.25 (3.08)	−0.39	0.25	19.38 (4.36)	−0.59	0.36
	Regularly	22.46 (3.70)	−0.49	0.77	20.44 (4.09)	−0.56	0.24
Social relationships (3 items; α = 0.66)	Never	9.15 (2.82)	−0.40	−0.77	9.70 (2.41)	−0.40	0.33
	Occasionally	10.58 (1.68)	−0.45	0.83	10.29 (2.61)	−0.61	−0.27
	Regularly	10.77 (2.46)	0.11	−0.85	10.80 (2.10)	−0.51	0.11
Environment (8 items; α = 0.75)	Never	22.23 (3.75)	0.28	−0.34	24.42 (4.18)	−0.17	−0.12
	Occasionally	24.75 (4.29)	−0.50	0.45	25.71 (4.53)	0.02	0.62
	Regularly	27.04 (5.16)	−0.03	−0.20	27.01 (4.28)	−0.12	0.71

**Table 4:** Pearson’s *r* coefficients between all scales calculated for participants practicing sports: below the diagonal AD group (*n* = 38), above the diagonal TD group (*n* = 214).

		1.	2.	3.	4.	5.	6.	7	8.	9.	10.	11.	12.	13.
1. WHOQOL – physical health	*r*	‒	0.62 **	0.49 **	0.55 **	0.42 **	0.52 **	0.48 **	−0.53 **	0.22 **	0.33 **	0.26 **	0.43 **	0.06
	*p*		< 0.001	< 0.001	< 0.001	< 0.001	< 0.001	< 0.001	< 0.001	.001	< 0.001	< 0.001	< 0.001	0.399
2. WHOQOL – psychological health	*r*	0.72 **	‒	0.65 **	0.52 **	0.54 **	0.77 **	0.74 **	−0.74 **	0.19 **	0.31 **	0.27 **	0.56 **	0.04
	*p*	< 0.001		< 0.001	< 0.001	< 0.001	< 0.001	< 0.001	< 0.001	.006	< 0.001	< 0.001	< 0.001	0.548
3. WHOQOL – social relationships	*r*	0.49 **	0.63 **	‒	0.49 **	0.39 **	0.60 **	0.50 **	−0.45 **	0.23 **	0.20 **	0.09	0.31 **	0.07
	*p*	.002	< 0.001		< 0.001	< 0.001	< 0.001	< 0.001	< 0.001	.001	.004	.192	< 0.001	0.330
4. WHOQOL – environment	*r*	0.64 **	0.57 **	0.41 *	‒	0.42 **	0.57 **	0.39 **	−0.35 **	0.19 **	0.29 **	0.20 **	0.32 **	0.04
	*p*	< 0.001	< 0.001	.011		< 0.001	< 0.001	< 0.001	< 0.001	.005	< 0.001	.004	< 0.001	0.597
5. GSE	*r*	0.44 **	0.66 **	0.58 **	0.17	‒	0.54 **	0.59 **	−0.38 **	0.26 **	0.18 **	0.27 **	0.49 **	0.23 **
	*p*	.006	< 0.001	< 0.001	0.297		< 0.001	< 0.001	< 0.001	< 0.001	0.008	< 0.001	< 0.001	0.001
6. SWLS	*r*	0.58 **	0.77 **	0.49 **	0.49 **	0.54 **	‒	0.65 **	−0.56 **	0.13	0.26 **	0.18 *	0.45 **	0.05
	*p*	< 0.001	< 0.001	0.002	0.002	< 0.001		< 0.001	< 0.001	.051	< 0.001	0.010	< 0.001	0.496
7. SEES – PWB	*r*	0.25	0.39 *	0.30	−0.001	0.53 **	0.45 **	‒	−0.63 **	0.32 **	0.19 **	0.22 **	0.51 **	0.10
	*p*	0.135	0.015	0.065	0.993	0.001	0.004		< 0.001	< 0.001	0.007	0.001	< 0.001	0.141
8. SEES – PD	*r*	−0.59 **	−0.62 **	−0.58 **	−0.48 **	−0.42 **	−0.54 **	−0.14	‒	−0.16 *	−0.28 **	−0.25 **	−0.53 **	−0.01
	*p*	< 0.001	< 0.001	< 0.001	.002	.009	< 0.001	0.412		0.018	< 0.001	< 0.001	< 0.001	0.868
9. I-TIPI-R Extraversion	*r*	0.32	0.28	0.34 *	−0.02	0.24	0.18	0.37 *	−0.29	‒	−0.09	0.02	0.13	0.16 *
	*p*	0.053	0.095	0.039	0.885	0.143	0.287	0.024	0.073		0.211	0.780	0.051	0.016
10. I-TIPI-R Agreeableness	*r*	−0.15	−0.11	−0.02	−0.04	0.04	−0.15	0.02	0.07	−0.14	‒	0.23 **	0.34 **	0.10
	*p*	0.356	0.529	0.929	0.803	0.794	0.363	0.901	0.687	0.404		0.001	< 0.001	0.137
11. I-TIPI-R Conscientiousness	*r*	0.30	0.33 *	0.04	0.08	0.45 **	0.14	0.36 *	−0.22	0.15	0.20	‒	0.25 **	−0.02
	*p*	0.066	0.042	0.797	0.636	0.005	0.394	0.025	0.191	0.376	0.220		< 0.001	0.806
12. I-TIPI-R Emotional stability	*r*	0.33 *	0.32 *	0.33 *	−0.07	0.50 **	0.22	0.16	−0.30	0.36 *	0.17	0.22	‒	0.04
	*p*	0.047	0.049	0.041	0.668	0.001	0.181	0.350	0.070	0.025	0.318	0.180		0.530
13. I-TIPI-R Openness	*r*	0.01	−0.04	0.07	−0.31	0.08	−0.14	0.12	−0.25	0.18	−0.001	0.28	0.21	‒
	*p*	0.977	0.834	0.660	0.059	0.627	0.414	0.472	0.130	0.270	0.994	0.094	0.202	

* Correlation is significant at the 0.05 level (2-tailed); ** Correlation is significant at the 0.01 level (2-tailed).

**Table 5:** Pearson’s *r* coefficients between all scales calculated for participants not practicing sports: below the diagonal AD group (*n* = 13), above the diagonal TD group (*n* = 56).

		1.	2.	3.	4.	5.	6.	7	8.	9.	10.	11.	12.	13.
1. WHOQOL – physical health	*r*	‒	0.66 **	0.52 **	0.55 **	0.33 *	0.63 **	0.38 **	−0.46 **	0.12	−0.04	−0.05	0.18	−0.06
	*p*		< 0.001	< 0.001	< 0.001	0.013	< 0.001	0.004	< 0.001	0.386	0.798	0.728	0.174	0.667
2. WHOQOL – psychological health	*r*	0.54	‒	0.74 **	0.57 **	0.44 **	0.77 **	0.60 **	−0.68 **	0.36 **	−0.09	0.13	0.42 **	0.08
	*p*	0.057		< 0.001	< 0.001	.001	< 0.001	< 0.001	< 0.001	0.007	0.517	0.334	0.001	0.560
3. WHOQOL – social relationships	*r*	0.23	0.29	‒	0.51 **	0.31 *	0.67 **	0.48 **	−0.53 **	0.12	0.11	0.10	0.34 *	0.04
	*p*	0.454	0.329		< 0.001	0.022	< 0.001	< 0.001	< 0.001	0.389	0.423	0.487	0.011	0.758
4. WHOQOL – environment	*r*	0.25	0.27	0.39	‒	0.24	0.56 **	0.39 **	−0.43 **	0.12	−0.08	−0.17	0.11	−0.06
	*p*	0.417	0.364	0.187		0.071	< 0.001	0.003	0.001	0.381	0.549	0.209	0.413	0.650
5. GSE	*r*	0.72 **	0.75 **	0.05	0.31	‒	0.46 **	0.60 **	−0.46 **	0.37 **	0.05	−0.05	0.24	0.18
	*p*	0.005	0.003	0.868	0.298		< 0.001	< 0.001	< 0.001	0.006	0.704	0.698	0.078	0.183
6. SWLS	*r*	0.71 **	0.55	0.24	0.42	0.68 *	‒	0.56 **	−0.57 **	0.20	0.03	−0.02	0.39 **	0.07
	*p*	0.006	0.051	0.430	0.159	0.011		< 0.001	< 0.001	0.141	0.805	0.908	0.003	0.597
7. SEES – PWB	*r*	0.77 **	0.14	−0.09	0.07	0.52	0.60 *	‒	−0.29 *	0.28 *	−0.06	−0.07	0.14	0.29 *
	*p*	0.002	0.645	0.770	0.834	0.068	0.029		0.028	0.036	0.664	0.622	0.292	0.032
8. SEES – PD	*r*	−0.65 *	−0.56 *	.10	−0.15	−0.61 *	−0.38	−0.67 *	‒	−0.36 **	−0.08	−0.13	−0.53 **	0.08
	*p*	0.017	0.048	0.744	0.627	0.028	0.197	0.012		0.006	0.572	0.352	< 0.001	0.569
9. I-TIPI-R Extraversion	*r*	0.00	0.40	−0.66 *	−0.09	0.50	0.01	−0.01	−0.40	‒	−0.14	−0.09	0.03	0.22
	*p*	1.000	0.174	0.015	0.759	0.080	0.981	0.967	0.178		0.309	0.516	0.832	0.102
10. I-TIPI-R Agreeableness	*r*	0.15	0.47	0.23	−0.28	0.16	0.002	−0.16	−0.18	0.22	‒	0.11	0.25	−0.06
	*p*	0.619	0.103	0.446	0.358	0.597	0.996	0.609	0.561	0.472		0.443	0.068	0.666
11. I-TIPI-R Conscientiousness	*r*	0.67 *	0.74 **	0.24	0.24	0.86 **	0.63 *	0.28	−0.35	0.35	0.40	‒	0.15	−0.24
	*p*	0.013	0.004	0.440	0.424	< 0.001	0.022	0.356	0.248	0.240	0.176		0.286	0.072
12. I-TIPI-R Emotional stability	*r*	0.63 *	0.34	−0.01	−0.08	0.63 *	0.66 *	0.81 **	−0.54	0.10	0.06	0.42	‒	−0.11
	*p*	0.020	0.259	0.981	0.804	0.022	0.015	0.001	0.055	0.753	0.839	0.148		0.432
13. I-TIPI-R Openness	*r*	0.55	0.54	−0.26	0.07	0.70 **	0.31	0.21	−0.46	0.66 *	0.29	0.72 **	0.15	‒
	*p*	0.052	0.056	0.396	0.828	0.008	0.302	0.497	0.114	0.014	0.336	0.006	0.622	

* Correlation is significant at the 0.05 level (2-tailed); ** Correlation is significant at the 0.01 level (2-tailed).
